# Genome Sequences of Two Pathogens of Cruciferous Crops, Xanthomonas campestris pv. raphani MAFF 106181 and *X. campestris* pv. campestris MAFF 301176

**DOI:** 10.1128/MRA.00887-20

**Published:** 2020-10-15

**Authors:** Takashi Fujikawa, Yasuhiro Inoue

**Affiliations:** a Institute of Fruit Tree and Tea Science, National Agriculture and Food Research Organization (NARO), Tsukuba, Ibaraki, Japan; b Central Region Agricultural Research Center, NARO, Tsukuba, Ibaraki, Japan; University of Arizona

## Abstract

Xanthomonas campestris pv. raphani and *X. campestris* pv. campestris are the causal agents of bacterial spot and black rot of crucifers (Brassicaceae), respectively. Both pathogens are threats in the cultivation of cruciferous crops such as cabbage. Here, we sequenced a strain of each of these pathogens.

## Abstract (precis)

Xanthomonas campestris pv. *raphani* and *X. campestris* pv. *campestris* are the causal bacteria of bacterial spot and black rot of crucifers (*Brassicaceae*), respectively. Both pathogens are threats in the cultivation of cruciferous crops such as cabbage. Here, we sequenced a strain of each of these pathogens.

## ANNOUNCEMENT

Xanthomonas campestris pv. raphani (synonym, X. campestris pv. armoraciae) is the pathogen responsible for bacterial spot of crucifers (1 – 3), and *X. campestris* pv. campestris is responsible for black rot of crucifers (4 – 6). Both pathogens have the potential to pose a global threat to the production of cruciferous (Brassicaceae) crops such as cabbage, broccoli, and Japanese radish. Since these are closely related bacteria, it is important to elucidate the common pathogenic genes and investigate the unique gene regions. Here, we report the complete genome sequence of a strain of *X. campestris* pv. raphani and a draft genome sequence of a strain of *X. campestris* pv. campestris.

As representative strains of *X. campestris* pv. raphani and *X. campestris* pv. campestris, we used MAFF 106181 (radish isolate) and MAFF 301176 (cabbage isolate), respectively, from the NARO Genebank (https://www.gene.affrc.go.jp/index_en.php). Both strains were recovered on potato-sucrose agar (PSA) medium from freeze-dried stocks, and these were cultivated in yeast-peptone (YP) broth at 27°C for 1 day with agitation at 140 rpm. Then, 1-ml aliquots of each culture were used for genomic DNA extraction with a Wizard genomic DNA purification kit (Promega, Madison, WI, USA). Genome sequencing of *X. campestris* pv. raphani and *X. campestris* pv. campestris was performed by the Beijing Genomics Institute (Shenzhen, China). In this study, default parameters were used for all software.

The *X. campestris* pv. raphani MAFF 106181 genome was *de novo* sequenced using the PacBio RS II platform (Menlo Park, CA, USA) after the sequence template was prepared from the *X. campestris* pv. raphani genomic DNA using the SMRTbell template prep kit v1.0 (Pacific Biosciences) according to the manufacturer’s protocol. Four single-molecule real-time (SMRT) cell zero-mode waveguide sequencing arrays were used to generate the PacBio subread set; subreads smaller than 1 kb were removed. The pbdagcon program (https://github.com/PacificBiosciences/pbdagcon) was used to self-correct the PacBio subreads. We obtained 297,824 subreads (average length, 10,819 bp; *N*_50_, 12,276 bp) in this sequence. Draft genomic unitigs were assembled from a set of high-quality corrected consensus sequence subreads (the unitig data were directly assembled with error correction to eliminate low-quality reads) by using the Celera Assembler software. Since both ends of the unitig were completely overlapped, we determined that this genome is circular. To improve the accuracy of the genome sequences, single-base corrections were made using GATK (Genome Analysis Toolkit, https://www.broadinstitute.org/gatk/) and SOAP tool packages (SOAP2, SOAPsnp, and SOAPindel).

The *X. campestris* pv. campestris MAFF 301176 genome was sequenced using the Illumina HiSeq 4000 platform (San Diego, CA, USA). Genomic DNA was sheared randomly by using a Bioruptor ultrasonicator (Diagenode, Denville, NJ, USA), and paired-end fragment libraries with read lengths of 500 bp were constructed using physicochemical methods with the BGI (Beijing Genomics Institute) kit. The libraries were sequenced, and raw reads of low quality (reads with a Phred score of ≤20 bases, a number of undetermined bases [*N*] of ≥10%, or very short length [≤5 bases]) were discarded. We obtained a total of 10,078,836 paired-end reads of 150 bp. The reads were assembled using SOAPdenovo v1.05.

The complete *X. campestris* pv. *raphani* genome and the draft *X. campestris* pv. campestris genome were annotated using the NCBI Prokaryote Genome Annotation Pipeline (PGAP) v4.3 (7).

The *X. campestris* pv. raphani MAFF 106181 genome consisted of a single circular chromosome of 4,942,039 bp with a GC content of 65.29%. The genome coverage was 652-fold. PGAP identified 4,269 genes, including 6 rRNA and 53 tRNA genes. The *X. campestris* pv. campestris MAFF 301176 draft genome consisted of 90 scaffolds (*N*_50_, 123,765 bp) covering a total of 5,123,729 bp with a GC content of 64.99%. The genome coverage was 997-fold. PGAP identified 4,414 genes, including 3 rRNA and 52 tRNA genes.

### Data availability.

Both the complete genome sequence of *X. campestris* pv. raphani MAFF 106181 (accession no. CP058243) and the draft genome sequence of *X. campestris* pv. campestris MAFF 301176 (accession no. JACAWT00000000) have been deposited in GenBank. The raw sequencing reads have been deposited under SRA accession no. SRR12108209 and SRR12108220, respectively.
